# Size Matters a Lot: Drought-Affected Italian Oaks Are Smaller and Show Lower Growth Prior to Tree Death

**DOI:** 10.3389/fpls.2017.00135

**Published:** 2017-02-21

**Authors:** Michele Colangelo, Jesús J. Camarero, Marco Borghetti, Antonio Gazol, Tiziana Gentilesca, Francesco Ripullone

**Affiliations:** ^1^School of Agricultural Forest Food and Environmental Sciences, University of BasilicataPotenza, Italy; ^2^Pyrenean Institute of Ecology – Consejo Superior de Investigaciones CientíficasZaragoza, Spain

**Keywords:** anisohydric species, drought-induced dieback, growth, mortality, *Quercus frainetto*, tree rings, wood anatomy

## Abstract

Hydraulic theory suggests that tall trees are at greater risk of drought-triggered death caused by hydraulic failure than small trees. In addition the drop in growth, observed in several tree species prior to death, is often interpreted as an early-warning signal of impending death. We test these hypotheses by comparing size, growth, and wood-anatomy patterns of living and now-dead trees in two Italian oak forests showing recent mortality episodes. The mortality probability of trees is modeled as a function of recent growth and tree size. Drift-diffusion-jump (DDJ) metrics are used to detect early-warning signals. We found that the tallest trees of the anisohydric Italian oak better survived drought contrary to what was predicted by the theory. Dead trees were characterized by a lower height and radial-growth trend than living trees in both study sites. The growth reduction of now-dead trees started about 10 years prior to their death and after two severe spring droughts during the early 2000s. This critical transition in growth was detected by DDJ metrics in the most affected site. Dead trees were also more sensitive to drought stress in this site indicating different susceptibility to water shortage between trees. Dead trees did not form earlywood vessels with smaller lumen diameter than surviving trees but tended to form wider latewood vessels with a higher percentage of vessel area. Since living and dead trees showed similar competition we did not expect that moderate thinning and a reduction in tree density would increase the short-term survival probability of trees.

## Introduction

Drought-induced tree mortality is becoming a major ecological concern as the planet becomes warmer and mortality episodes increase worldwide (Allen et al., 2010). Nevertheless, many research gaps still exist concerning the patterns of drought-related tree death and the reason why some trees are more prone to die than other coexisting individuals (McDowell et al., 2008). This has fostered the investigation of dieback mechanisms caused by hydraulic failure (substantial xylem embolism) and carbon starvation (carbon losses greatly exceed carbon gains) (Sevanto et al., 2014). In addition, the search for early-warning signals of tree death, particularly using retrospective proxies, such as tree-ring or wood-anatomy data, has been stimulated in connection with dieback mechanisms (Dobbertin, 2005; Camarero et al., 2015; Cailleret et al., 2016; Pellizzari et al., 2016).

In this study, we focus on such early-warning signals of tree death and we model the mortality probability as a function of recent growth and tree height. Further, we focus on a sub-Mediterranean ring-porous oak (*Quercus frainetto*) because in the Allen et al. (2010) global review, only 11% of the mortality cases involved similar oak species, which are the dominant hardwood trees in many drought-prone areas. In addition, Bennett et al. (2015) report in their review that, drought-related mortality increased with tree height in 65% of the cases examined, where few or none involved oak species.

Published reviews (Bennett et al., 2015; McDowell and Allen, 2015) have lead to predict that tall and dominant trees of isohydric species (characterized by strict control of water loss by closing stomata) are at greater risk of dying due to heat and drought stress; whereas smaller trees of anisohydric species (with less control of water loss and high stomatal conductance rates, i.e., showing variable maximum values of xylem tension depending on changes in soil water availability and vapor pressure deficit - hereafter VPD; cf. Klein, 2014) are predicted to better survive under drought conditions (McDowell and Allen, 2015). This does not agree with some field data showing that anisohydric species as oaks are not able to tolerate severe droughts showing growth decline and increased mortality rates (Klos et al., 2009).

In theory, anisohydric is expected to be linked to an enhanced hydraulic conductivity (Martínez-Vilalta and Garcia-Forner, 2016). However, recent studies show that there is a continuum between isohydric and anisohydric behavior rather than a clear threshold between these two functional strategies (Klein, 2014). Therefore, we can expect that coexisting oak trees display different wood-anatomical features reflecting contrasting xylem hydraulics and vulnerabilities to drought-induced dieback, connected with more or less anisohydric behavior (Rosner et al., 2016).

In trees the probability of tree death is usually related to size (height or diameter) (Grote et al., 2016), since empirical evidence shows that small trees are slow growing and have higher death probability than bigger ones (Monserud and Sterba, 1999; Das et al., 2007; Holzwarth et al., 2013). Overall, tree size, growth rate, and competition seem reasonable predictors of tree mortality (Cailleret et al., 2016). Some mortality models use relative growth rates to consider the influence of tree size (sometimes relative to the neighboring trees’ size) on the likelihood of tree death; whilst other models directly employ tree diameter and rarely height as predictors of mortality since these are the most widely measured variables in forestry (Yao et al., 2001; Ruiz-Benito et al., 2013). However, few studies have addressed if tree size is related to the probability of tree death after dry spells (but see Camarero et al., 2016).

In addition to static variables, such as tree size, mortality models are usually formulated as a function of growth rates or trends (based either on ring-width or on basal area increment – BAI) calculated over time periods (often between 5 and 50 years) prior to tree death (Pedersen, 1998; Wyckoff and Clark, 2000; Bigler and Bugmann, 2003, 2004; Bigler et al., 2004; Bircher et al., 2015). In selecting the growth variable, BAI reflects well long-term changes in tree vigor (Phipps and Whiton, 1988; Duchesne et al., 2003), and it is a better proxy of changes in biomass increment than tree-ring width (Bowman et al., 2013). However, establishing robust growth-mortality associations is challenging because dead and decaying trees can present elevated growth rates prior to tree death (Wunder et al., 2008; Levanic et al., 2011; Voltas et al., 2013; Sangüesa-Barreda et al., 2015). Mortality models depend on the selection and calculation of explanatory growth variables, such as growth rates or growth trends (see a recent review by Cailleret et al., 2016). Therefore, since growth and tree height are closely related, a biologically meaningful model of tree mortality should consider both variables.

In the specific case of oaks, low rates of radial growth and negative growth trends often precede tree death (Pedersen, 1998, 1999; Drobyshev et al., 2007; Stojanović et al., 2015), as it happens in other tree species (Dwyer et al., 1995; Ogle et al., 2000; Haavik et al., 2011; Gea-Izquierdo et al., 2014; Kane and Kolb, 2014; Mamet et al., 2015). However, a high year-to-year variability of growth, the occurrence of abrupt growth declines or an increase in lag-1 autocorrelation have also been associated to high mortality rates (Suarez et al., 2004; Das et al., 2007; Camarero et al., 2015). In addition, increased growth sensitivity to climate stressors (e.g., drought severity) has recently been detected in dead trees as compared with living trees (Linares and Camarero, 2012).

In this study, we employ tree size, growth, and wood-anatomy data and estimate recent competition intensity to understand if drought differently affected mortality rates in coexisting individuals of two decaying Mediterranean oak (*Q. frainetto*) forests. Specifically, we quantified wood anatomy to evaluate if dead trees formed a less xylem with more potential hydraulic conductivity characterized by latewood vessels with wider lumen areas (corresponding to a more anisohydric behavior during the dry summer season) than surviving trees; whilst, these latter should produce a xylem characterized by vessels with narrower lumen areas. Moreover, we employed a modeling approach to investigate mortality probability as a function of recent growth and tree size. We hypothesize those taller trees with wider vessels will be more prone to drought-induced tree death than smaller trees forming vessels with narrower lumen.

## Materials and Methods

### Study Sites

We selected two forests showing recent drought-induced canopy dieback and mortality located near the San Paolo Albanese (40° 01′ 20′ N, 16° 20′ 46′ E, 950 m a.s.l., mean slope 25–30%; hereafter SP site) and Oriolo (40° 00′ 10″ N, 16° 23′ 30″ E; 770 m a.s.l., mean slope 25%; hereafter OR site) villages situated in the Basilicata region, southern Italy. The SP site is a pure high forest located in the Pollino National Park and it has a density of 348 stems ha^-1^. The mean diameter at 1.3 m (dbh) and age are 40 cm and 145 years, respectively. The OR site is also a high forest with density of 444 stems ha^-1^, and mean dbh and age are 35 cm and 138 years. The soil in both study sites is sandy or silty clay. No silvicultural treatment has been applied for the past 40 years.

Since the early 2000s both forests presented trees with dieback symptoms (shoot dieback, leaf loss and withering, growth decline, high mortality, and epicormic shoot formation).

### Climate and Drought Data

Climate in the study area is Mediterranean characterized by dry and warm summers (summer precipitation is 79 mm) and wet and mild winters (winter precipitation is 257 mm) with mean annual temperature of 16.4°C and annual precipitation of 742 mm (data from Oriolo station, 40° 03′ 11″ N, 16° 26′ 47″ E, 445 m a.s.l., 1950–2015 period). The warmest and coldest months are July (mean maximum temperature of 33.5°C) and January (mean minimum temperature of 4.0°C), respectively, whereas the driest and wettest months are July (22 mm) and December (99 mm). Drought occurs from June to September.

To evaluate drought-growth associations since 1950 we downloaded the Standardized Precipitation Evapotranspiration Index (SPEI) for the 0.5° grid where the study sites are located using the Global SPEI database webpage^1^. The SPEI is a multiscalar drought index, i.e., it expresses monthly cumulative drought stress at different time scales (e.g., a 10-month May SPEI considers drought stress from previous August to May). The SPEI considers the effects of temperature and evapotranspiration on drought severity and indicates wet (positive SPEI values) and dry (negative SPEI values) conditions (Vicente-Serrano et al., 2010).

### Study Species

The Italian oak (*Q. frainetto* Ten.) is native to southern Italy, the Balkans, and north-west Turkey. It is a winter deciduous, shade-intolerant and sub-Mediterranean oak species forming ring-porous wood and reaching heights of at least 30 m (Chatziphilippidis and Spyroglou, 2004). Radial growth of the Italian oak is very sensitive to dry conditions in late spring and summer (Sanders et al., 2014).

### Field Sampling and Competition Index

We sampled couples of coexisting and dominant living and dead trees located at less than 20 m apart within each couple, and sampled at the SP and OR sites. Firstly, seven and four circular plots (radius of 15 m) were randomly located in the SP and OR sites, respectively, to obtain estimates of density of dead trees at each site. Recently dead trees were characterized by the almost complete absence of green leaves or the presence of dead shoots and brown leaves remaining in the crown. In the SP site, 24 couples randomly selected of living and dead trees were sampled (*n* = 24 living and *n* = 24 dead trees), whilst at the OR sites 18 couples were sampled plus additional six living trees (*n* = 24 living and *n* = 18 dead trees). First, the tree dbh and height of each tree were measured using dbh tapes and a laser rangefinder, respectively. Second, to study growth and wood anatomy we extracted three increment cores per tree at breast height (1.3 m) separated by 120° using a Pressler increment borer. Two cores were used for dendrochronological analyses and the remaining core was used for wood anatomy.

The recent competition experienced by each focal tree *i* was calculated as a function of the tree size (DBH) and proximity of the three nearest *j* neighbors following Forrester et al. (2013) and using the following formula:

$${CI}_{i}=\overset{3}{\underset{j=1}{\Sigma }}\left(\frac{{BA}_{j}}{d_{i,j}}\right)$$

where *CI_i_* is the competition intensity experienced by the focal tree *i*, BA*_j_* is the basal area of neighboring *j* trees, and *d_ij_* is the distance between the tree *i* and the *j* neighbors. We assume that choosing the three nearest neighbors provides a reasonable estimate of the tree-to-tree competition in the neighborhood of the focal trees since the samples stands correspond to relatively open high forests where most trees are dominant.

### Tree-Ring Data

Wood samples were air-dried and the surface of the cores was cut using a sledge core microtome (Gärtner and Nievergelt, 2010). Tree rings were visually cross-dated and tree-ring widths were measured to the nearest 0.01 mm using a binocular microscope coupled to a computer with the LINTAB package (Rinntech, Heidelberg, Germany). To estimate tree age at 1.3 m, when a core did not reach the pith, the total missing width and the number of missing rings were estimated by interpolating the distance to the pith using the curvature of the innermost rings of the sample. The COFECHA program (Holmes, 1983) was used to evaluate the visual cross-dating of tree-ring series. To quantify growth we transformed the tree-ring widths into BAI using the following formula:

$$BAI=\pi \left(R_{t}^{2}-R_{t-1}^{2}\right)$$

where *R* is the radius of the tree and *t* is the year of tree–ring formation.

Since BAI values are often not normally distributed, we used the median. In addition, we calculated: the BAI coefficient of variation (CV) as a measure of its dispersion; the slope of the BAI linear trend with time (Trend); and the BAI lag-1 autocorrelation (A1). Following Cailleret et al. (2016), these variables were calculated for several periods representing long, mid and short-term BAI changes previous to tree death: 1980–2014 (35 years prior to tree death), 2000–2014 (15 years prior to tree death), and 2005–2014 (10 years prior to tree death).

### Wood Anatomy

We selected five couples of living-dead trees per site to perform wood-anatomical analyses. These trees were selected because they show the highest correlations between their BAI series and the mean BAI series of living or dead trees from each site. Wood anatomy was analyzed for the 1980–2013 period because several dead trees did not form the complete 2014 tree ring. Transversal sections (thickness of 20 mm) were prepared from each core tree by dividing it into pieces of approximately 2 cm length. Sections were cut using a sliding microtome (Microm HM 400, Thermo Sci., Walldorf, Germany) and then stained with safranin (1%) and astra blue (2%), dehydrated with ethanol (70, 95, and 100%) and xylol, and fixed on microscope slides using Eukitt^®^ mounting medium. Images were captured at 20–40 × magnification using a light microscope (Zeiss Axiophot, Carl Zeiss Microscopy, Jena, Germany). Earlywood (EW) and latewood (LW) vessels were analyzed in tangential windows of 2 and 0.3 mm, respectively. EW vessels were considered those with lumen diameters larger than 50 mm. Transversal vessel diameters (along the radial direction) and areas were measured using the ImageJ software for image analysis (Schneider et al., 2012). We obtained the following wood-anatomical variables following Scholz et al. (2013): ring area, EW and LW areas, absolute and relative (%) areas occupied by vessels in the EW and LW, EW and LW vessel areas (mean, minimum, and maximum values), EW and LW vessel density. We also calculated the hydraulic diameter (*Dh*) for all vessels measured within each ring by weighting individual conduit diameters to correspond to the average Hagen–Poiseuille lumen theoretical hydraulic conductivity for a vessel size (Tyree and Zimmermann, 2002). The *Dh* was calculated as the average of S*d*^5^/S*d*^4^, where *d* is the diameter of each vessel (Sperry et al., 1994).

### Statistical Analyses

Differences between living and dead trees in tree features (size, age, and competition index), growth and wood anatomy were assessed using Mann–Whitney *U* tests. The relationships between BAI and the SPEI were evaluated using Pearson correlations considering a 32-year long period previous to the dieback onset (1950–1981) and another period of similar duration encompassing the dieback process (1982–2013). Following Hentschel et al. (2014), we used the Wilcoxon signed-rank test to check if growth (BAI) and wood-anatomical variables differed between living and dead trees. To compare BAI-SPEI relationships between living and dead trees we used Mann–Whitney *U* tests. We chose these non-parametric tests because they are robust against deviations from standard distributions (Gibbons and Chakraborti, 2011).

### Logistic Models of Tree Mortality

We evaluated if tree mortality is related to short and long-term growth characteristics by examining the variables of the selected logistic regression models. Generalized linear models (GLMs) with binomial error distribution (i.e., logistic regressions) were used to predict the survival probability of a tree *i* at time *t*, hereafter labeled *Pr* (*Y_i,t_* = 1), and to analyze the corresponding growth-mortality relationships (Bigler and Bugmann, 2004). Logistic regression models with fixed effects were used as trees were growing at one single site and most of them died on the same year following this equation:

$$\log\left[\frac{\mathrm{Pr}\left(Y_{i,t}=1\right)}{\left(1-\mathrm{Pr}\left(Y_{i,t}=1\right)\right)}\right]=\alpha_{0}+\beta_{0}+\delta_{0}\times G_{i,t,p}$$

where Pr (*Y_i,t_*) follows a binomial distribution with *Y_i,t_* = 0 indicating that tree *i* is dead at time *t*, while *Y_i,t_* = 1 indicates that the tree is alive; α_0_ is the intercept, β_0_ are tree variables (height, dbh, age); and δ_0_ is the coefficient for growth variables calculated over a period of length *p* (*G_i,t,p_*).

Models were fitted using a combination of several variables. In the SP site, the evaluated models included the following variables height, dbh, age, *CIi* and variables derived from BAI (BAI_2005-2014_, CV BAI_2005-2014_, BAI Trend _1980-2014_, and BAI A1_2005-2014_). In the OR site, the evaluated models included the following variables height, dbh, age, *CIi* and the following variables derived from BAI (BAI_2005-2014_, CV BAI_1980-2014_, BAI Trend _2000-2014_, and BAI A1_2005-2014_).

Model performance was assessed using an information-theoretic approach (Burnham and Anderson, 1998). We calculated Akaike information criterion (AIC), the change in AIC for each model relative to the best model (DAIC), and Akaike weights (*Wi*) which evaluate the relative importance of the model. These metrics are commonly used to assess model performance, but the AIC is strongly positively correlated to the number of samples used to calibrate the model, and depends on the equitability of the sampling (Lawson et al., 2014). This is problematic when comparing the performance of models fitted on different sample sizes as in this study. Discrimination metrics may be more suitable in this case because they reflect the ability to separate survival and death observations (Lawson et al., 2014). Here, we employ the Area Under the Receiver Operating Characteristic Curve (AUC) which is not dependent on the equitability of the sampling. AUC values range from 0 to 1, with 0.5 indicating a random model. A model providing an excellent prediction and showing a notable amount of discrimination has AUC ≥ 0.9 (Fielding and Bell, 1997; Hosmer et al., 2013). Selected models had the highest *Wi* and AUC values. We present the three models with the highest *Wi*. We presented the AUC of the best-fitted model and its McFadden pseudo-*R*^2^ which is a likelihood ratio comparing a model without any predictor to a model including all predictors (Hosmer et al., 2013). GLMs were fitted using the function “glm” of the software R (R Core Team, 2015), and their AUC was calculated using the package *ROCR* (Sing et al., 2013).

### Non-parametric Drift-Diffusion-Jump Metrics

Since BAI series provide a summary of the tree growth history, characteristics of these BAI series (Dakos et al., 2012) can be investigated to detect early-warning signals of tree death (Camarero et al., 2015). A sharp growth decline is often a discrete indicator of portending tree death (Pedersen, 1998), but BAI series represent continuous data whose underlying processes are unknown, possibly nonlinear and occur at short and long-term scales (Kane and Kolb, 2014). In the case of growth data, critical transitions may be detected using tools as non-parametric drift-diffusion-jump (DDJ) metrics which fit a general DDJ model as a surrogate for the unknown nonlinear processes generating the data (Brock and Carpenter, 2012; Mamet et al., 2015). Using these methods, estimates of the drift (deterministic) and diffusion (stochastic) components of underlying processes, as well as an indicator of the conditional variance, can be computed (Brock and Carpenter, 2012). Briefly, these DDJ metrics include: the drift which measures instantaneous changes due to deterministic trends corresponding to local rates of change; the diffusion which measures the standard deviation of relatively small shocks that occur every year; the jumps which are large intermittent shocks due to uncorrelated changes; the conditional variance that rises to infinity at a critical point and it is estimated as the difference between the second conditional moment and the square of the first conditional moment; and the total variance which combines the contributions of diffusion and jumps (Carpenter and Brock, 2011). The DDJ model is able to assess both high- and low-frequency patterns in BAI data estimating the probability of a range shift (Mamet et al., 2015). We expect rises in the conditional and total variances and a decrease in diffusion and an increase in jump intensity prior to a sharp BAI drop portending tree death. Following Mamet et al. (2015), we calculated the DDJ metrics on log+1-transformed BAI series for dying and living trees at both study sites. In this regard, the 1970–2014 period was considered, when BAI started stabilizing in terms of growth and age. The DDJ metrics were calculated with the *earlywarnings* R package (Dakos et al., 2012).

## Results

### Differences in Tree Size between Living and Dead Trees and Mortality Incidence

**FIGURE 1:**
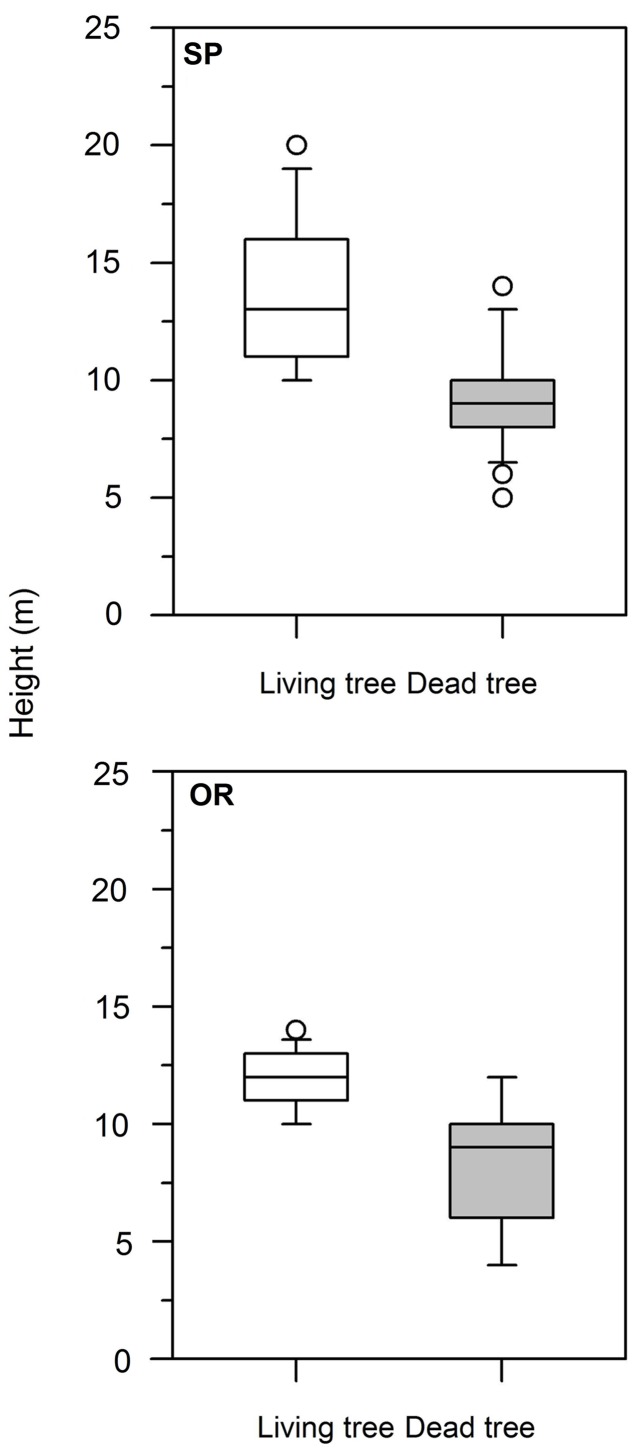
**Box plots showing the significant differences in tree height between living (white boxes) and dead (gray boxes) Italian oak trees at the SP and OR study sites**.

At both study sites, dead trees presented lower height than living trees (**Table 1**; **Figure 1**). At SP site, this difference was also observed in dbh, whereas the rest of the analyzed variables (age, *CIi*) did not show significant differences. Considering dead trees, dbh and height were not significantly related neither at the SP site (*r* = 0.31, *P* = 0.14) nor at the OR site (*r* = 0.38, *P* = 0.07). In contrast, in living trees dbh and height were significantly related at both study sites (SP, *r* = 0.70, *P* = 0.0001; OR, *r* = 0.62, *P* = 0.006).

**Table 1:** Characteristics of the living (L) and recently dead (D) oak trees sampled in the two study sites (OR and SP).

Site	Tree type	No. trees	Dbh (cm)	Height (m)	Age at 1.3 m (years)	Competition index
SP	L	24	32.6 ± 0.9b	14.1 ± 0.9b	146 ± 2a	657.5 ± 45.1a
	D	24	28.2 ± 1.0a	9.5 ± 0.5a	143 ± 2a	708.4 ± 71.2a
OR	L	24	29.1 ± 0.7a	11.6 ± 0.3b	141 ± 2a	603.6 ± 51.9a
	D	18	27.5 ± 1.5a	8.7 ± 0.7a	139 ± 3a	536.2 ± 76.6a


*Values are means ± SE. Significant differences (*P* < 0.05; Mann–Whitney *U* tests) between living and dead trees within each site are indicated by different letters.*

**FIGURE 2:**
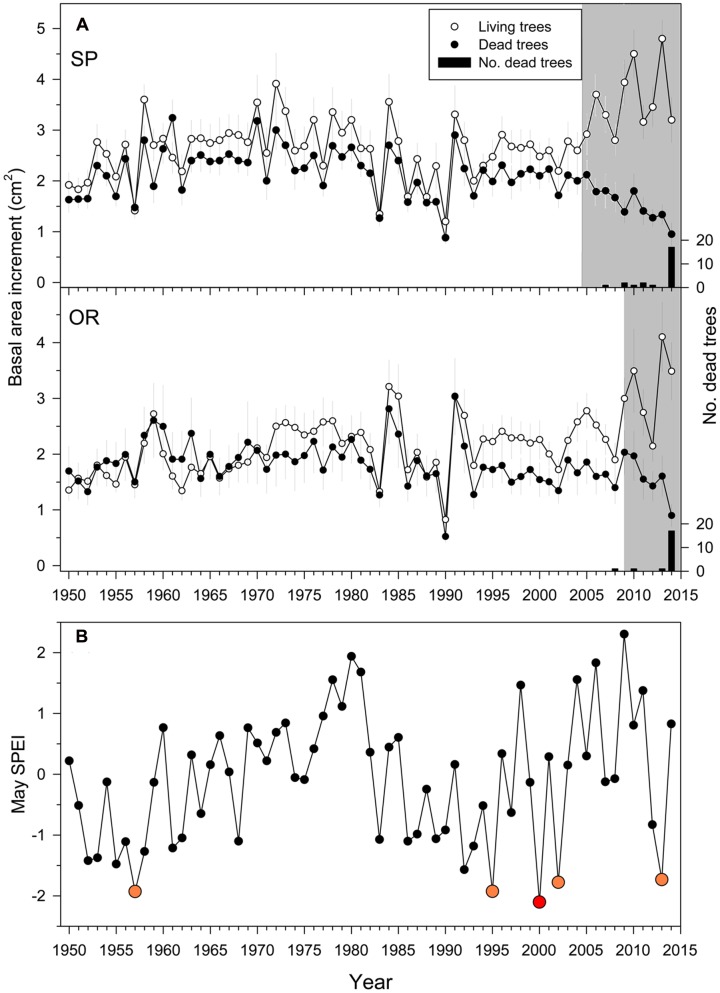
**Growth** **(A)** quantified as basal area increment (BAI) of living (white symbols) and dead (filled symbols) Italian oak trees at the SP and OR study sites and growing-season drought severity **(B)**. In the uppermost plots, the black bars show the number of dead trees classified according to their last formed ring which was considered the death year (right *y* axes). The drought severity was quantified using the May SPEI drought index calculated at a 10-month long scale (big red and yellow symbols show very dry years; the 2000 year is highlighted with a red symbol because it showed the driest May). The gray filled areas indicate periods when the BAI of dead trees was significantly (*P* < 0.05) lower than that of living trees Values are means ± SE.

The densities of dead trees at the SP and OR sites were 35 (8%) and 25 stems ha^-1^ (5%), respectively, suggesting a greater impact of drought at SP than at OR site. Overall, 70 and 83% of the trees sampled at the SP and OR sites, respectively, died in 2014 (**Figure 2**).

### Growth Patterns and Trends of Living and Dead Trees

Trends in BAI were always significantly lower in dead than in living trees regardless of the compared period and the study site; such differences were magnified from 2000 onwards (**Table 2**). Observed BAI values corresponded to narrow rings and low growth rates (SP, mean ring width of 0.79 mm; OR, mean ring width of 0.72 mm) At both sites, median BAI values were also lower in dead than in living trees for the 2000–2014 and 2005–2014 periods. The BAI CV of living trees from the OR site was higher than in dead trees for all periods, except in the shortest one (2005–2104). Lastly, at the SP site, BAI A1 was higher in living than in dead trees during the 1980–2014 period but it was more negative in living trees when only the shorter period from 2000 onwards was considered.

**Table 2:** Selected values of basal-area increment of living (L) and recently dead (D) trees sampled at the two study sites (SP and OR) and calculated for three different periods (1980–2014, 35 years prior to tree death; 2000–2014, 15 years prior to tree death; and 2005–2014, 10 years prior to tree death).

Site	Tree type	1980–2014				2000–2014				2005–2014			
				
		Median (cm^2^)	CV (%)	Trend (cm^2^ year^-1^)	A1	Median (cm^2^)	CV (%)	Trend (cm^2^ year^-1^)	A1	Median (cm^2^)	CV (%)	Trend (cm^2^ year^-1^)	A1
SP	L	2.72 ± 0.44a	49.8 ± 2.3a	0.02 ± 0.01b	0.27 ± 0.04b	3.16 ± 0.30b	40.6 ± 2.2a	0.04 ± 0.01b	-0.11 ± 0.05a	3.38 ± 0.18b	40.8 ± 2.5a	0.04 ± 0.01b	-0.32 ± 0.04a
	D	1.99 ± 0.36a	47.3 ± 2.8a	-0.01 ± 0.01a	0.12 ± 0.05a	1.80 ± 0.23a	52.6 ± 6.1a	-0.02 ± 0.01a	0.09 ± 0.07b	1.54 ± 0.13a	66.4 ± 10.7a	-0.04 ± 0.02a	-0.01 ± 0.09b
OR	L	2.27 ± 0.30a	57.0 ± 2.9b	0.02 ± 0.01b	0.16 ± 0.04a	2.52 ± 0.29b	53.3 ± 3.8b	0.05 ± 0.01b	0.02 ± 0.05a	2.76 ± 0.20b	54.8 ± 5.2a	0.05 ± 0.01b	-0.14 ± 0.06a
	D	1.65 ± 0.39a	43.9 ± 2.7a	-0.01 ± 0.01a	0.06 ± 0.05a	1.60 ± 0.24a	43.3 ± 4.3a	-0.01 ± 0.01a	0.03 ± 0.08a	1.60 ± 0.20a	48.4 ± 7.5a	-0.02 ± 0.01a	0.02 ± 0.09a


*Presented variables are: median, coefficient of variation (CV), linear trend and the lag-1 autocorrelation (A1). Significant differences (*P* < 0.05; Mann–Whitney *U* tests) between living and dead trees within each site are indicated by different letters. Values are means ± SE.*

The BAI differences in single years between living and dead trees were significant from 2005 onwards at the SP site, and from 2009 onwards at the OR site (**Figure 2A**). Since then, BAI dropped in dead trees with some BAI increases corresponding to wet years (2009–2010) and steadily increased in living trees. Note that the divergence in BAI between living and dead trees started after severe spring droughts, which occurred in 1995, 2000, 2002, and 2013 (**Figure 2**). According to the calculated 10-month long May SPEI, droughts of similar severity since 1950 only occurred before in 1957 (**Figure 2B**).

### Drought-Growth Relationships in Living and Dead Trees

**FIGURE 3:**
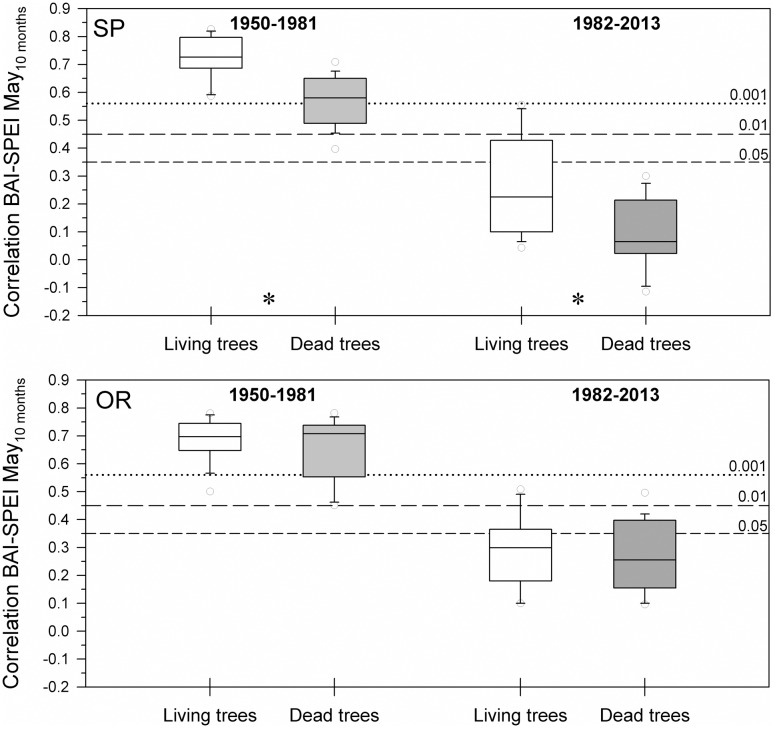
**Drought-growth associations differed between living and dead trees at the SP site but not at the OR site.** The box plots show the correlations between BAI and the May SPEI drought index calculated at 10-months long time scales. Correlations were calculated for 32-year long periods prior (1950–1981) and overlapping (1982–2013) with the drought-induced growth decline and increase in mortality rate of the studied Italian oaks. Asterisks indicate significant (*P* < 0.05) differences found between living and dead trees at the SP site. Empty and filled boxes correspond to living and dead trees, respectively. The dashed and dotted horizontal lines indicate significance levels.

The positive correlations between BAI and the 10-month long May SPEI were significantly higher in living than dead trees at the SP site for the 1950–1981 and 1982–2013 periods, i.e., during a period previous to the dieback onset and during the dieback process (**Figure 3**). Contrastingly, these differences were not significant at the OR site. Overall, living trees from the SP site showed higher BAI increases to wet conditions than living trees from the OR site.

### Wood Anatomy

**FIGURE 4:**
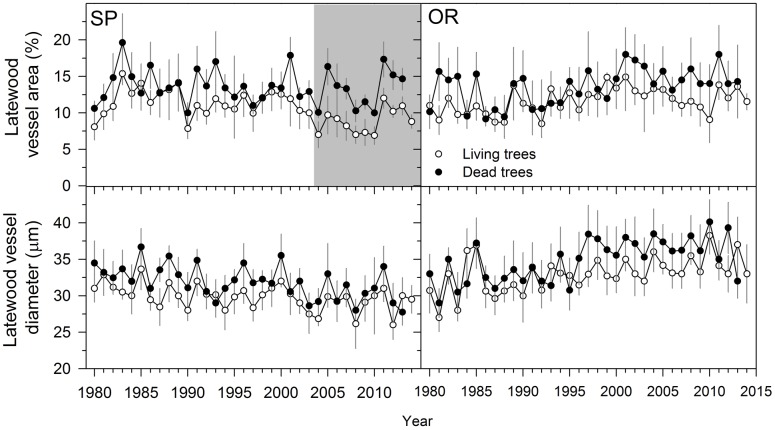
**Latewood anatomical variables measured in living (empty symbols) and dead (filled symbols) Italian oak trees at SP and OR study sites.** The gray filled areas indicate periods when the variables significantly (*P* < 0.05) differ between living and dead trees (Wilcoxon signed-rank test). Since most dead trees did not form the 2014 ring we did not present values for them and for this year. Values are means ± SE.

When comparing living and dead trees, we found no significant differences neither in hydraulic terms (*Dh*) nor in EW anatomical features (**Table 3**). However, LW vessel diameter tended to be wider for dead trees at the SP site (**Table 3**; **Figure 4**). In both sites, the LW vessel density of dead trees was also reduced as compared with living trees and the percentage of LW area occupied by vessels increased in now-dead trees.

**Table 3:** Wood-anatomical variables obtained for living (L) and recently dead (D) trees in the two study sites (SP and OR).

Site	Tree type	No. measured vessels	*Dh*, hydraulic diameter (mm)	Earlywood			Latewood		
					
				Vessel diameter (mm)	Vessel area (%)	Vessel density (No. mm^-2^)	Vessel diameter (mm)	Vessel area (%)	Vessel density (No mm^-2^)
SP	L	14668	349.2 ± 3.0a	264.4 ± 2.5a	39.1 ± 1.0a	8 ± 1a	30.0 ± 0.3a	12.4 ± 0.4a	175 ± 5b
	D	8532	354.1 ± 3.9a	267.4 ± 3.4a	37.6 ± 1.1a	6 ± 1a	31.4 ± 0.3b	13.7 ± 0.5b	160 ± 7a
OR	L	15824	348.9 ± 3.0a	265.0 ± 2.6a	38.7 ± 1.1a	7 ± 1a	33.0 ± 0.5a	12.2 ± 0.4b	139 ± 4b
	D	10536	345.8 ± 4.2a	267.0 ± 3.0a	36.7 ± 1.6a	5 ± 1a	34.0 ± 0.6a	13.3 ± 0.5a	119 ± 4a


*Data correspond to the 1980–2014 period and are presented as means ± SE.*

*Significant differences (*P* < 0.05; Mann–Whitney *U* tests) between living and dead trees within each site are indicated by different letters.*

### Logistic Models of Tree Mortality: Tree Height Matters

The selected models indicated that the probability of tree death increased at both sites for trees with lower height and presenting a sharper growth reduction (BAI trend) for the 2000–2014 period. At the SP site, the most parsimonious model also selected a lower median BAI for the 2005–2014 period, associated to an increasing chance of mortality; whereas at the OR site, the lower BAI CV, which occurred during the 1980–2014 period, was also selected as a characteristic of dead trees (**Tables 2** and **4**).

**Table 4:** Logistic regression results of the top three performing logistic models of tree survival for Italian oaks sampled at each of the two study sites (SP, OR) based on tree age, size (DBH, height) and growth characteristics.

Site	Model	DAIC	*Wi*	AUC	McFadden pseudo *R*^2^
SP	Height^∗∗^ + Trend_2000-2014_^∗^ + BAI_2005-2014_^∗^	2.21	0.76	0.983	0.77
	Height + Trend_2000-2014_ + BAI_2005-2014_ + DBH	4.51	0.24		
	Height + Trend_1980-2014_	16.47	0.01		
OR	Trend_2000-2014_^∗∗^ + Height^∗^ + CV_1980-2014_	0.64	0.47	0.949	0.60
	Trend_2000-2014_ + Height + DBH + CV_1980-2014_	0.96	0.40		
	Trend_2000-2014_ + Height	3.13	0.13		


*Reported statistics area: DAIC (differences in the Akaike information criterion), *Wi* = Akaike weights, AUC = area under the receiver operating characteristic curve, McFadden pseudo-*R*^2^. The AUC and McFadden pseudo-*R*^2^ are only presented for the best models. Growth characteristics were calculated considering basal area increment (BAI) for three periods: 1980–2014 (1–35 years before tree death), 2000–2014 (1–15 years before tree death), and 2005–2014 (1–10 years before tree death). These characteristics are: the median (BAI), the CV, the linear slope of BAI with time (Trend); and the BAI lag-1 autocorrelation (A1). Note that A1 was not selected in none of the models. Asterisks indicate significance values of the variables for the selected model with highest *Wi* and AUC values: ^∗^*P* < 0.05; ^∗∗^*P* < 0.01.*

### Early-Warning Signals of Tree Death: DDJ Metrics of BAI Series

**FIGURE 5:**
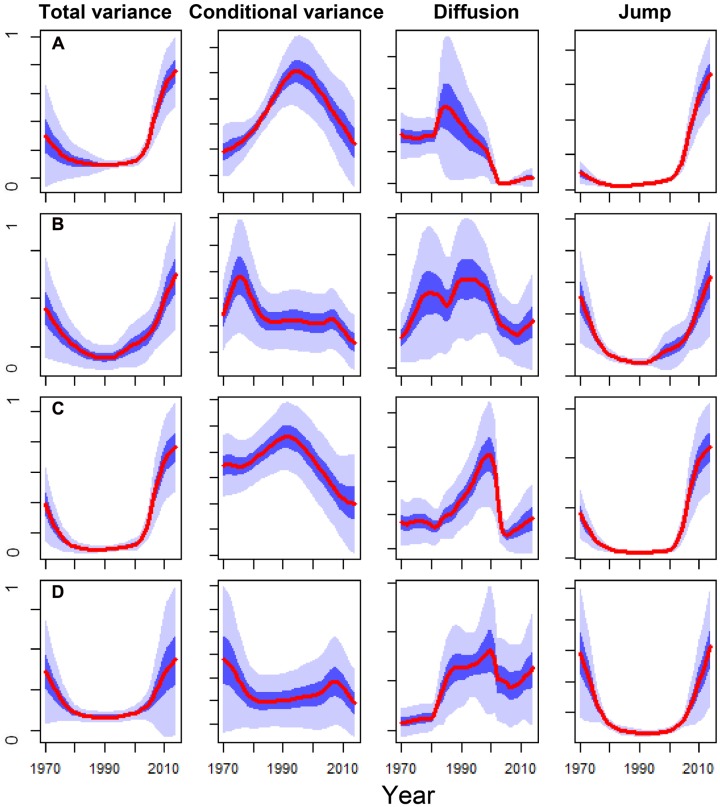
**Comparison of the main non-parametric drift-diffusion-jump (DDJ) metrics calculated for the basal-area increment series (1970–2014 period) of the Italian oak dead trees sampled at the SM** **(A)** and OR **(C)** sites vs. the living trees from SM **(B)** and OR **(D)** sites. The DDJ metrics include the total variance, the conditional variance, the diffusion and the jump. The red lines show the variance medians among trees and light and dark blue shadings indicate the 10–90% and 40–60% confidence intervals, respectively. The *y* axes show variance components and they were standardized to the 0–1 range.

The DDJ metrics of the dead trees from the SP site showed a peak of conditional variance around 1995 and a sharp decrease in diffusion in the early 2000s. This coincided with an increase in total variance and jump intensity; whereas living trees did not present similar patterns, particularly concerning conditional variance and diffusion (**Figure 5**). The conditional variance of dead trees from the OR site peaked in the early 1990s and the diffusion fell around 2005, whilst living trees did not show these changes in DDJ metrics (**Figure 5**). The years when diffusion rapidly dropped in the case of dead trees roughly coincided with those when BAI of living trees was significantly higher than BAI of now-dead trees (compare **Figures 2A** and **5**).

## Discussion

In contrast with Bennett et al. (2015), we observed that now-dead trees were smaller than living trees (**Table 1**), which agrees with a previous report on dieback of Mediterranean oaks showing a smaller size and a greater crown leaf loss (Camarero et al., 2016). This difference in height may have predisposed to the conspicuous and rapid leaf loss of recently dead trees associated to apical shoot dieback (F. Ripullone, personal observation).

Our findings do not imply that height and above ground competition are the most relevant variables to predict tree death in similar drought-prone sites. Alternatively, belowground competition for water and nutrients could constrain the vertical development of the trees most vulnerable to drought. Tree size *per se* (i.e., diameter; height; leaf, stem, and root volumes) might be more important to explain long-term changes in tree vigor (Mencuccini et al., 2005). Since tree diameter and height often covary, a bigger sample size may be helpful in future studies to discern the most relevant explanatory variable (diameter or height) of tree mortality.

The smaller size of dead trees and the lack of differences in the lumen diameter of the wider earlywood vessels between living and dead trees (**Table 3**) lead to refute our main hypotheses. It is likely that initial genetic or phenotypic differences or contrasting topoedaphic restrictions bring to distinct capacities of height and root depth among coexisting trees. Larger and taller trees with deeper root systems are able to explore more soil volume, especially in sandy or silty soils, which can make them less vulnerable to intense and prolonged drought conditions.

However, in contrast with our results, Bennett et al. (2015) in their global review report that tall trees have higher mortality rates than small ones. According to this review, larger trees are at higher risk of drought-induced hydraulic failure because they experience greater resistance in transporting water upwards (i.e., higher potential gradient, conduit resistance). Drought-induced dieback preferentially affected tall Neotropical tree species because large trees were more vulnerable to drought, due to their lower capacity to buffer the negative effects of soil water deficit (Zhang et al., 2009). Therefore, associations between tree height and the probability of death do not seem to be so simple. In addition, many drought-induced dieback cases involve dry regions (Allen et al., 2010), where trees mainly compete for water (e.g., southwestern USA, southern Europe) and do not grow as tall as in wet sites where resources abound (e.g., California redwood forests). Obviously, Italian oak trees are not expected to show similar limitations to those experienced by very tall redwood trees, whose leaves and shoots show increasing turgor limitation with increasing height, even without soil moisture deficit (Woodruff et al., 2004). Our findings indicate that in drought-prone areas where trees do not become very tall as in most Mediterranean oak forests, a small height is a drawback to withstand dry spells.

The best-performing models of tree mortality did not select the competition index, in agreement with the similar values found for living and dead trees (**Tables 1** and **4**). In boreal, subalpine and temperate forests, a curvilinear U-shaped relationship between tree mortality and size (usually dbh) has often been observed, indicating that a small tree size relative to the stand neighbors increases the probability of mortality due to competition for light (Monserud and Sterba, 1999; Yao et al., 2001; Chen et al., 2008). Since big and old dying trees are often absent on those mortality-size analyses, the negative associations between mortality rate and tree size are also expected to occur in mature forests showing advanced successional stages (Oliver and Larson, 1996). This could be applied to the presented study case, where both sites were relatively old forests (**Table 1**). In temperate forests, competition for light strongly influences the growth of small trees, whereas competition for nutrients affects trees of diverse sizes (Coomes and Allen, 2007). Under those conditions, the small size and low growth rates of dying trees usually correspond to suppressed trees forming the understorey (Kobe et al., 1995; Wyckoff and Clark, 2002). However, in more open stands from dry areas, the competition for water and nutrients overrides this competition for light and affects growth rates and tree mortality (Linares et al., 2010). In the study drought-prone area, aboveground competition, in terms of radial growth, did not seem to drive tree death.

Considering whole-tree water storage capacity (capacitance) aids to explain our findings because the contribution of water stored in the stem (sapwood and bark) to canopy transpiration during dry periods is considerable and increases with tree size (Phillips et al., 2003). Therefore, the time to reach critical levels of drought stress should increase with tree size, as stem volume increases relative to leaf area (Scholz et al., 2011). This suggests that larger trees should be less susceptible to drought-triggered mortality than smaller trees as we found. Other studies have also found a larger size of living trees as compared with now-dead coexisting trees, as in this study. For instance, in the case of aspen forests, living trees showed a larger size than trees which did not survive (Hanna and Kulakowski, 2012; but see Worrall et al., 2008). In a Bishop pine forest in southern California, tree survivorship increased for the tallest trees, because they intercept more fog than smaller trees, thus counteracting drought stress (Carbone et al., 2012; Baguskas et al., 2014). A simplified soil-plant-atmosphere-continuum model, applied on decaying piñon pines in southwestern USA, predicted that smaller trees were more prone to die after drought, which was in agreement with field observations of larger surviving pines on steep slopes where soil water content is low (Gentine et al., 2016). The pattern of larger size in living than in dead trees would correspond to a higher growth rate in living rather than dead trees, i.e., prior to the stress-induced dieback, which is opposite to what has been observed in some dieback studies (Levanic et al., 2011; Voltas et al., 2013).

Regarding growth data, dead trees showed lower growth rates than living trees 10 years prior to tree death at both study sites, albeit differences were significant 5 years prior to death (**Figure 2**). Our findings agree with previous studies indicating that a reduced growth in the 5–50 years preceding death is a reliable predictor of tree mortality (e.g., Pedersen, 1998; Ogle et al., 2000; Bigler and Bugmann, 2004; Das et al., 2007; Hereş et al., 2012; Ireland et al., 2014). We also observed that the growth of dead trees was less reduced by drought stress than living trees at the most affected SP site (**Figure 3**), suggesting that dead trees were less sensitive to water shortage. This lower responsiveness of dead trees prior to the dieback onset could be explained by their lower growth rates or because their growth was more constrained by other site (poor access to soil water due to a less developed root system) or climatic factors (warmer summer temperatures; see Camarero et al., 2016). The uncoupling between growth and the SPEI drought index observed since 1982 in SP dead trees could indicate that other climatic stressors (e.g., warmer temperatures) override the effects of water deficit on growth or that stressed trees are losing responsiveness to water deficit. Contrastingly, increased early growth rates of dead trees have been described in subalpine conifers, which were characterized by a direct association between fast early growth, large size and decreased longevity (Bigler and Veblen, 2009). In other studies, trees dying after dry spells showed intensified growth responsiveness to water shortage, associated to canopy dieback and to a lower whole crown gas exchange relative to living trees (McDowell et al., 2010; Hereş et al., 2012).

The severe 2000s droughts triggered an irreversible growth decline in the dead oak trees (**Figure 2**). The usefulness of such sharp or gradual drops in growth after drought, as early-warning signals of tree death, has been proven either by utilizing widely employed tools, such as logistic models (**Table 4**) or non-parametric DDJ metrics (**Figure 5**). The maximum of conditional variance occurred prior to the drop in diffusion (**Figure 5**) which, according to Dakos et al. (2012), indicates the onset of a negative growth trend and could be used to determine the trend timespan in the logistic model. Accordingly, the selected mortality models included the growth trend for the 2000–2014 period (**Table 4**). Therefore, the drop in diffusion coincides with the timespan of BAI selected by the model. This suggests that both methods could be combined, i.e., DDJ could help to predefine the relevant timespans for logistic mortality models.

One methodological caveat of tree-ring data is their retrospective base. For example, it is possible that part of the sharp BAI increase of living trees observed during the 2010s was a growth release caused by a reduced competition for water and nutrients with dying trees (**Figure 2A**). This could represent a limitation of the sampling method based on the selection of “living-dead tree” couples, which assumes that neighboring trees are subjected to similar environmental constraints through time (e.g., competition); but tree death could generate a biased growth response of surviving trees. A similar release effect was observed in tropical forests, where drought caused a preferential death of large trees, which were more vulnerable to hydraulic deterioration. This lead to a competitive release of small suppressed trees whose growth rates increased after big trees died (Rowland et al., 2015). In that tropical forest, taller trees were predisposed to greater atmospheric water demand and loss in xylem functionality due to their longer hydraulic path lengths (Mencuccini et al., 2005); whereas smaller trees could avoid hydraulic deterioration through leaf water uptake. However, this seems not to be the case of the sub-Mediterranean oak forests subjected to seasonal droughts and reaching much lower heights than tropical trees.

Since dead trees did not form earlywood vessels with wider lumen areas than surviving trees, but tended to form wider latewood vessels occupying more xylem area (**Table 3**), their xylem would not be more vulnerable to cavitation. The severe droughts during the early 2000s occurred mainly in spring, but the latewood was more affected because in Mediterranean ring-porous oaks the enlargement of earlywood vessels depends more on winter temperature conditions prior to their formation, whereas the development of latewood is driven by spring to summer cumulative water deficit (Alla and Camarero, 2012). It could be described as a “latewood-biased” xylem providing more hydraulic conductivity during the mid to late growing season (Corcuera et al., 2006), and possibly corresponding to a more anisohydric behavior. An increase in VPD caused by warmer conditions, as those observed at the study area during the recorded 2000s droughts (**Figure 2B**), could have triggered a decrease in the leaf transpiring surface, which is a way to reduce the canopy evaporative water loss (DeLucia et al., 2000). However, such a sharp decrease in leaf biomass did not avoid tree death, which started to occur in 2007 and 2008 (**Figure 2A**). At the shoot level, winter-deciduous Mediterranean oaks often show a reduction in transpiring leaf area as compared with similar oak species from temperate areas. However, their anisohydric behavior, particularly when VPD is not elevated and soil water is available, may compensate for the loss in carbon gain caused by a lower leaf area (Peguero-Pina et al., 2016). In addition, Mediterranean anisohydric oaks such as *Quercus faginea* present premature leaf withering in response to drought. This reduction in carbon gain has been linked to xylem hydraulic failure (Peguero-Pina et al., 2015). Leaf loss could thus be seen as a mechanism of drought avoidance in winter-deciduous Mediterranean species such as the Italian oak. This punctual reduction in carbon uptake could be offset by elevated gas-exchange rates, but also by high primary-growth rates, allowing a rapid development of shoots and leaves in spring (Alla et al., 2012). Further research could also compare the patterns of growth, as related to those of carbon storage (non-structural carbohydrates) in coexisting Mediterranean oak species, showing drought-induced dieback. A higher dependence of carbohydrates stored in leaves could make some evergreen oak species (e.g., *Quercus ilex*) very sensitive to dry spells, whilst deciduous species could tolerate drought by preferentially storing carbohydrates in shoot wood (Camarero et al., 2016).

## Conclusion

The probability of oak death was negatively related to tree height, which is contrary to what hydraulic theory predicted. We interpret this result as increased drought sensitivity of small individuals compared to tall trees, suggesting that tall trees are better able than small trees to obtain soil water with their deeper root system under intense and prolonged drought stress. The more intense leaf shedding in recently dead oaks characterized the dieback process, which started during the early 2000s, when a strong decline in BAI began. This growth decline occurred about 10 years before most declining oaks died in 2014. The presented mortality models indicate that growth prior to tree death can be used as early-warning signal of impending tree mortality. Considering forest management, tree-to-tree competition did not predispose to tree death. In temperate forests competition in dense stands can contribute to drought-induced mortality (Pedersen, 1998), but at dry sites forest patches with low tree density also show high mortality rates (Dorman et al., 2015). This observation questions the validity of moderate thinning as a tool to increase the survival probability of trees facing prolonged droughts (McDowell et al., 2006), and indicates that intense thinning may be a better tool in some cases (Calev et al., 2016).

## Author Contributions

MC, JC, MB, TG, and FR conceived the idea and contributed to the writing of research project. MC, TG, and FR contributed to the field work. The dataset was analyzed by JC, MC, and AG. All authors contributed to the interpretation of the results. JC and MC wrote the first draft of the manuscript; thereafter all authors revised the first draft by rewriting, discussing and commenting. All authors read and approved the final draft.

## Conflict of Interest Statement

The authors declare that the research was conducted in the absence of any commercial or financial relationships that could be construed as a potential conflict of interest.

## Footnotes

1 http://sac.csic.es/spei/database.html
